# Insufficiency Fracture of the Proximal Femur in a Spastic Paraplegic Patient: A Case Report

**DOI:** 10.7759/cureus.80574

**Published:** 2025-03-14

**Authors:** Ibrahim S ElMaghrby, Ahmed Mahmoud, Deyaa Hammad

**Affiliations:** 1 Orthopaedics and Trauma, Ahmed Maher Teaching Hospital, Cairo, EGY; 2 Orthopaedic Surgery, Prince Mutaib Bin Abdulaziz Hospital, Al-Jouf, SAU; 3 Orthopaedics and Trauma, King Salman Hospital, Riyadh, SAU; 4 Orthopaedics and Trauma, Al-Azhar University, Cairo, EGY; 5 Orthopaedic Surgery, Mansoura Specialized Hospital, Mansoura, EGY

**Keywords:** femoral fracture, fragility fracture, insufficiency fracture, intramedullary nail, spastic paraplegia, spinal cord injury, subtrochanteric fracture femur

## Abstract

Managing lower extremity insufficiency fractures in patients with severe spastic paraplegia presents unique challenges. These fractures occur without significant trauma due to factors such as decreased bone quality and reduced muscle mass. The optimal treatment approach remains unclear due to the rarity of reported cases in the literature. This case report describes a severely displaced proximal femur fracture in a 33-year-old male with severe spastic paraplegia, mimicking a posterior hip dislocation deformity. The fracture was successfully treated with open reduction and internal fixation using a static intramedullary nail. This case underscores the importance of individualized treatment strategies for complex fractures in patients with spinal cord injuries and severe spastic paraplegia.

## Introduction

In the United States, there are approximately 285,000 individuals, mostly males, who live with spinal cord injury (SCI). Traumatic events, including falls, motor accidents, and acts of violence, are the most common reasons for SCI, resulting in neurological impairments that can be further complicated by various associated conditions. Motor vehicle accidents are a leading cause of SCI, accounting for 38.4% of all SCI cases [1]. The first six months following the injury are critical, as they have the highest percentage of bone loss; then, after 12-16 months, it starts to stabilize after a 30% decrease in bone mass [2]. However, the loss does not stop, and a gradual decline continues over time, reaching 50% loss after 10 years [3]. SCI patients are at risk of lower extremity fractures without any significant trauma as a secondary complication, often during simple activities such as repositioning or during physiotherapy and range of motion (ROM) exercises [4]. Osteoporosis-related bone density loss increases fracture susceptibility, with the distal femur and tibia being most affected, followed by the proximal femur and spine [5].

Efforts should be made to optimize the treatment of these fractures to minimize associated complications. However, regarding choosing between surgical and non-surgical interventions, there is a limited amount of published literature discussing the occurrence of proximal femur insufficiency fractures in spastic paraplegic patients. Furthermore, there is no unanimous consensus among medical teams, and the decision often varies depending on specific factors. This report attempts to give a summary of our experience using a static intramedullary nail (IMN) to treat a fragile, severely displaced proximal femoral fracture in a spastic paraplegic patient who required surgery after suffering thoracic vertebral fractures of the T7, T8, T9, and T10 vertebrae 15 years ago. Bilateral sublaminar claw hook stabilization from T4 to T10, connected by a single cross connector, was the surgical procedure. The patient involved in this case was informed about the data being submitted for publication and provided consent.

## Case presentation

**Figure 1:**
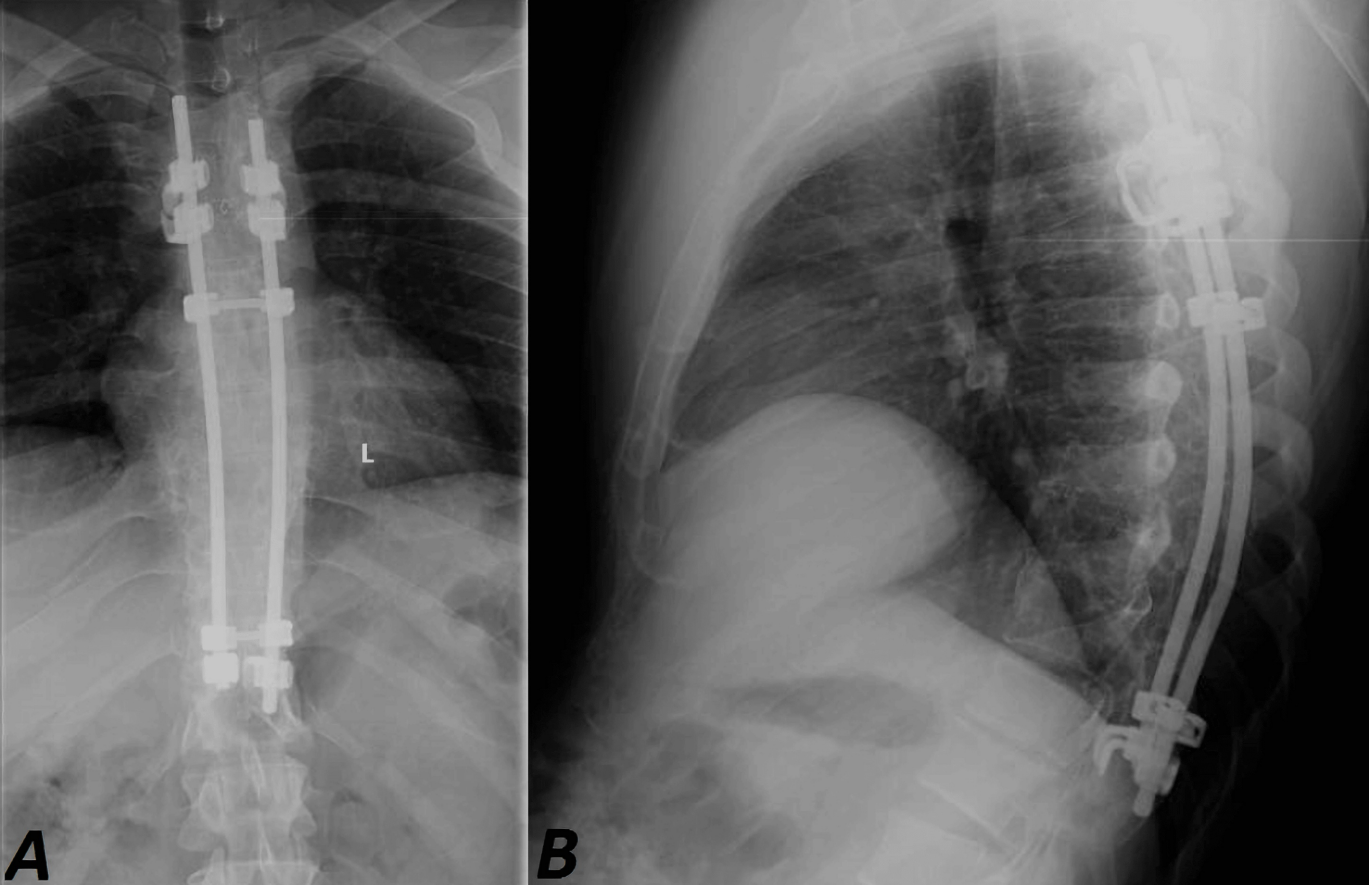
(A) Anteroposterior and (B) lateral radiographs show bilateral sublaminar wiring with proximal and distal claw hook stabilization from T4 to T10.

A 33-year-old man with spastic paraplegia presented to our emergency department with complaints of left lower limb pain and deformity after experiencing a sudden audible click while repositioning in bed. A history of spastic paraplegia 15 years prior was revealed, due to a motor vehicle accident, which resulted in bilateral posterior hip fracture dislocation that was treated conservatively. Additionally, the patient suffered thoracic vertebral fractures of T7, T8, T9, and T10 vertebrae, necessitating surgical intervention. The surgical intervention involved bilateral sublaminar wiring with proximal and distal claw hook stabilization from T4 to T10 (Figures 1A-1B). There were no medical comorbidities in the patient.

Upon examination, the patient presented with swelling, ecchymosis, crepitus, and a pronounced left thigh deformity resembling an acquired posterior hip dislocation. Additional signs included sweating, muscle spasticity, midplantar flexion of the feet, and flexion, adduction, and internal rotation of the hips and knees.

**Figure 2:**
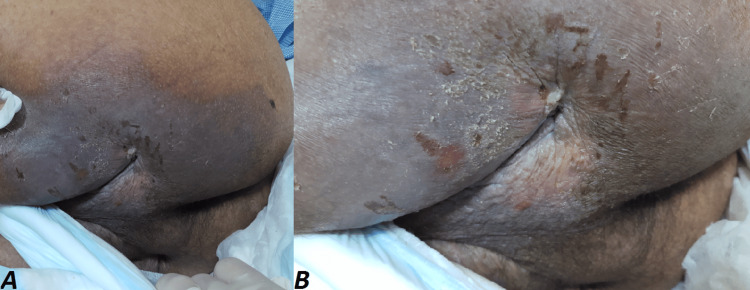
Clinical picture of healed bilateral severe ischial decubiti, presenting as large, deep dimples on the posterior surface of the upper thighs.

Despite treatment, due to severe degenerative osteoarthritic hips, the patient did not experience a significant improvement in his neurological conditions, and he experienced severe muscle spasticity in the lower limbs. This resulted in progressive muscle contractures, significantly impairing his mobility over the next 14 years. As a result, frequent physical therapy and rehabilitation sessions were performed, and there were no associated fractures in upper or lower limbs. However, perineal hygiene was compromised. Approximately one year prior, the patient was admitted to another hospital with a diagnosis of bilateral severe ischial decubiti. Over the last year, their condition improved, resulting in large, deep dimples bilaterally present on the posterior surface of the upper thighs (Figures 2A-2B).

**Figure 3:**
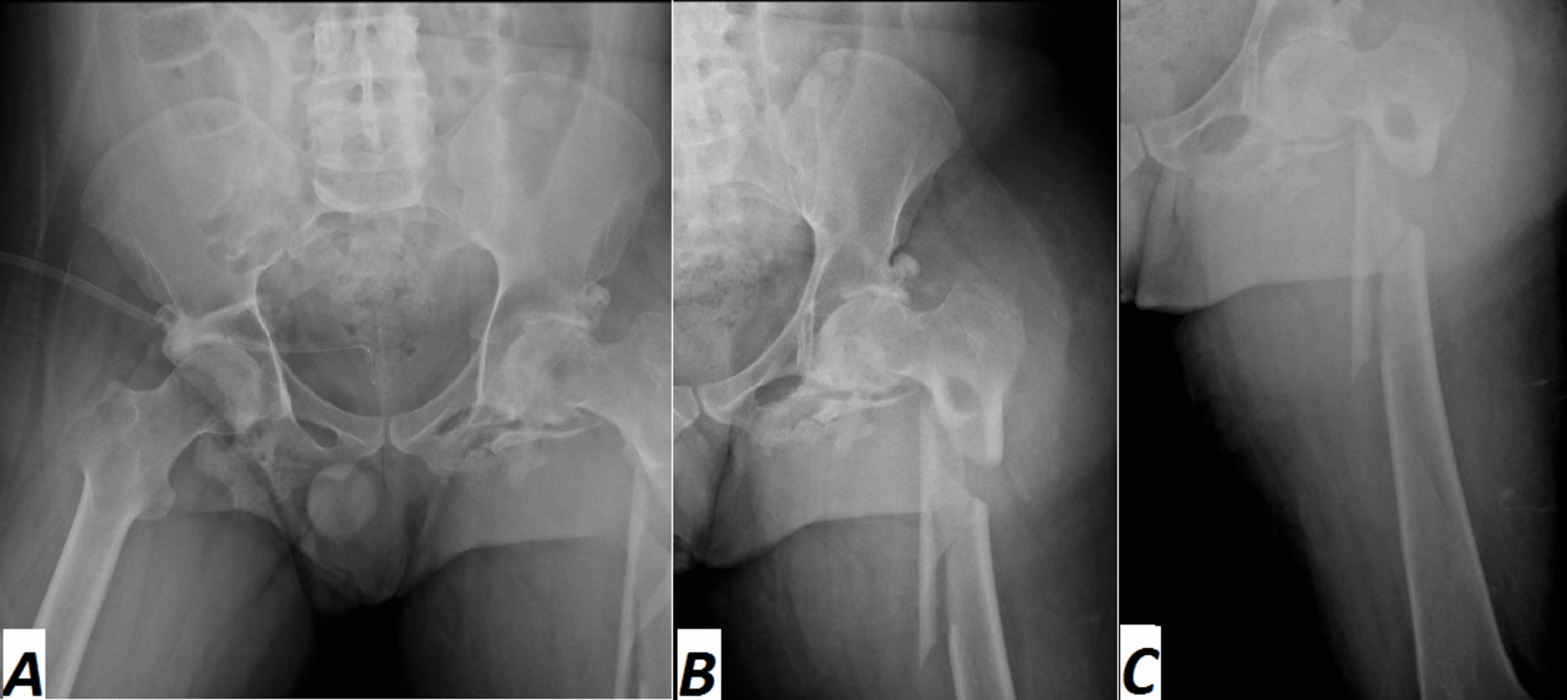
Preoperative (A) anteroposterior and (B) lateral radiographs show a closed, severely displaced, comminuted subtrochanteric left femur fracture.

He developed a bedsore on the left side, requiring daily dressing changes. Additionally, his suprapubic catheter was replaced one month ago. The patient presented with pronounced vitamin D insufficiency (vitamin D level: 7 ng/ml), low hemoglobin, and decreased ferritin levels of 10 ng/ml, 8.5 g/dl, and 15 mcg/L, respectively, and normocytic normochromic anemia. In addition, prealbumin level, blood culture, total lymphocyte count, and inflammatory markers known as nutritional evaluations were conducted. Extremity radiography films indicated a closed severely displaced comminuted subtrochanteric left femur fracture (Figures 3A-3C).

**Figure 4:**
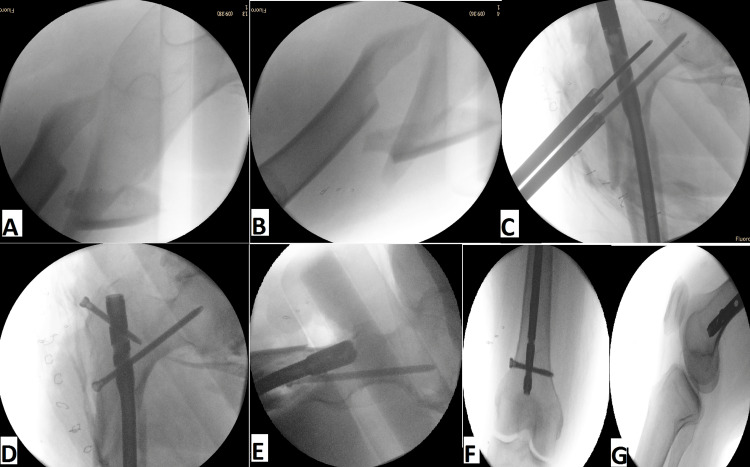
Intraoperative anteroposterior and lateral radiographs (A, B) of the proximal femur show a severely displaced subtrochanteric femur fracture. Anteroposterior and lateral radiographs (C, D, E, F, G) of the left femur show the intraoperative steps of intramedullary nail insertion.

At the time of hospitalization, his medications included oral vitamin D, a vitamin B12 supplement, a baclofen 10 mg tablet, an IV iron infusion, and Clexane 40 mg administered subcutaneously once daily. Open reduction and static intramedullary nail internal fixation were performed on the patient. However, setting up the patient on the traction table was the most challenging step due to the patient's inability to fully extend hip and knee joints. Hence, the patient was positioned with slight hip adduction and mid-flexion of both hip and knee. The patient received a dose of prophylactic antibiotics during general anesthesia. After preparing and draping, the proximal femur was exposed using a lateral approach. Nonetheless, the intraoperative reduction of the fracture and insertion of the guide wire was accompanied by some difficulty due to different factors, such as the proximal fragment's 90-degree flexion, muscular contracture, and degenerative osteoarthritis of the hip and knee joints, which was considered the second challenging step (Figure 4).

**Figure 5:**
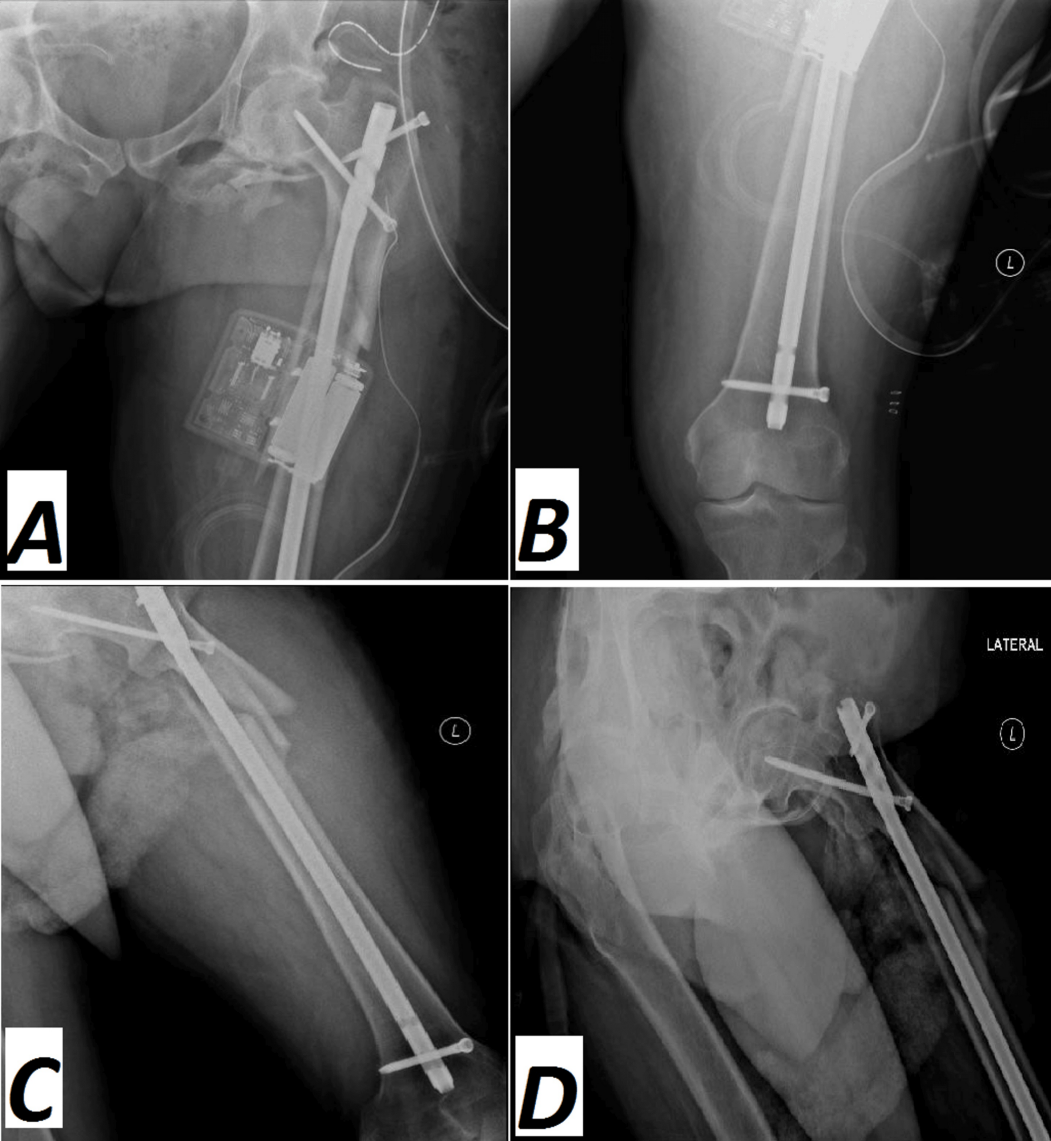
Immediate postoperative anteroposterior and lateral radiographs (A, B, C, D) of the left femur show a reduced, severely comminuted subtrochanteric fracture, fixed by an antegrade intramedullary nail.

After releasing the tensor fascia, we were able to achieve reduction and inserted a cephalic reconstructive nail using conventional methods. Because of the significant muscle spasm, adjusting the reconstructive screws proved challenging. During insertion, minimal proximal migration of the inferior screw in the neck was observed, prompting the placement of a short superior screw through the greater trochanter. Following wound irrigation, the tensor fascia lata muscle was repaired, a hemovac drain was inserted, and the surgical incision was sutured. Throughout the procedure, the patient remained hemodynamically stable. An immediate postoperative plain X-ray had been taken, showing a reduced severely comminuted subtrochanteric fracture fixed by an antegrade intramedullary nail (Figure 5).

**Figure 6:**
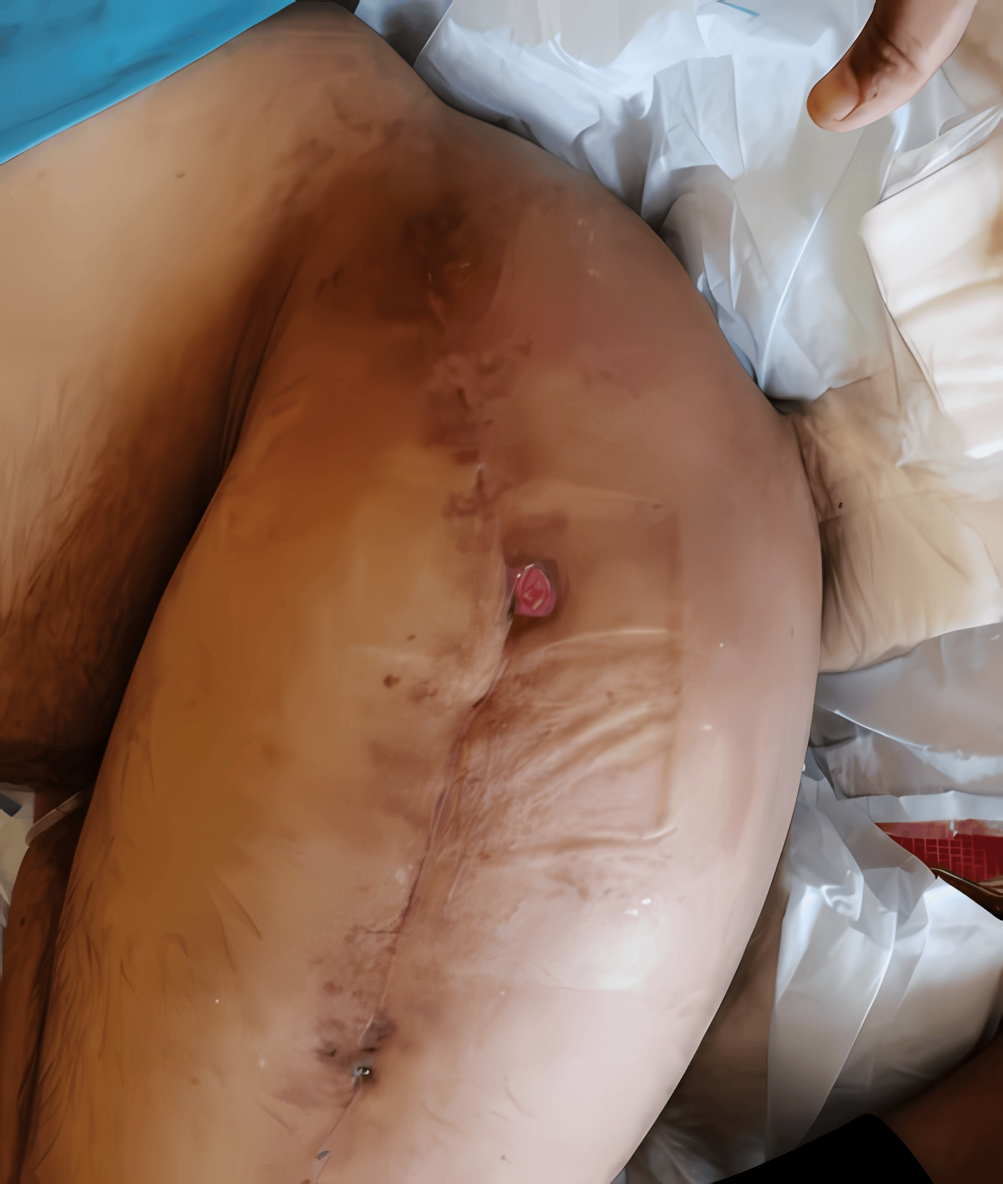
Clinical picture of the lateral aspect of the left thigh showing a small raw area at the skin incision site.

The patient was closely monitored in the postoperative phase, during which physical therapy was initiated. He was started on vancomycin 1 gram q12 hr intravenously for seven days, which was monitored by vancomycin level every other day, due to a history of acute bilateral ischial decubiti, along with intravenous iron, subcutaneous teriparatide, and vitamin D supplementation. Additionally, regular repositioning every two hours was essential for his care. Upon discharge, vitamin D, Clexane, and ciprofloxacin 500 mg bid were prescribed to the patient. No complications were observed, and the patient remained clinically stable except for a small raw area at the skin incision site, increased sweating, and muscle spasms, especially near the wound (Figure 6).

**Figure 7:**
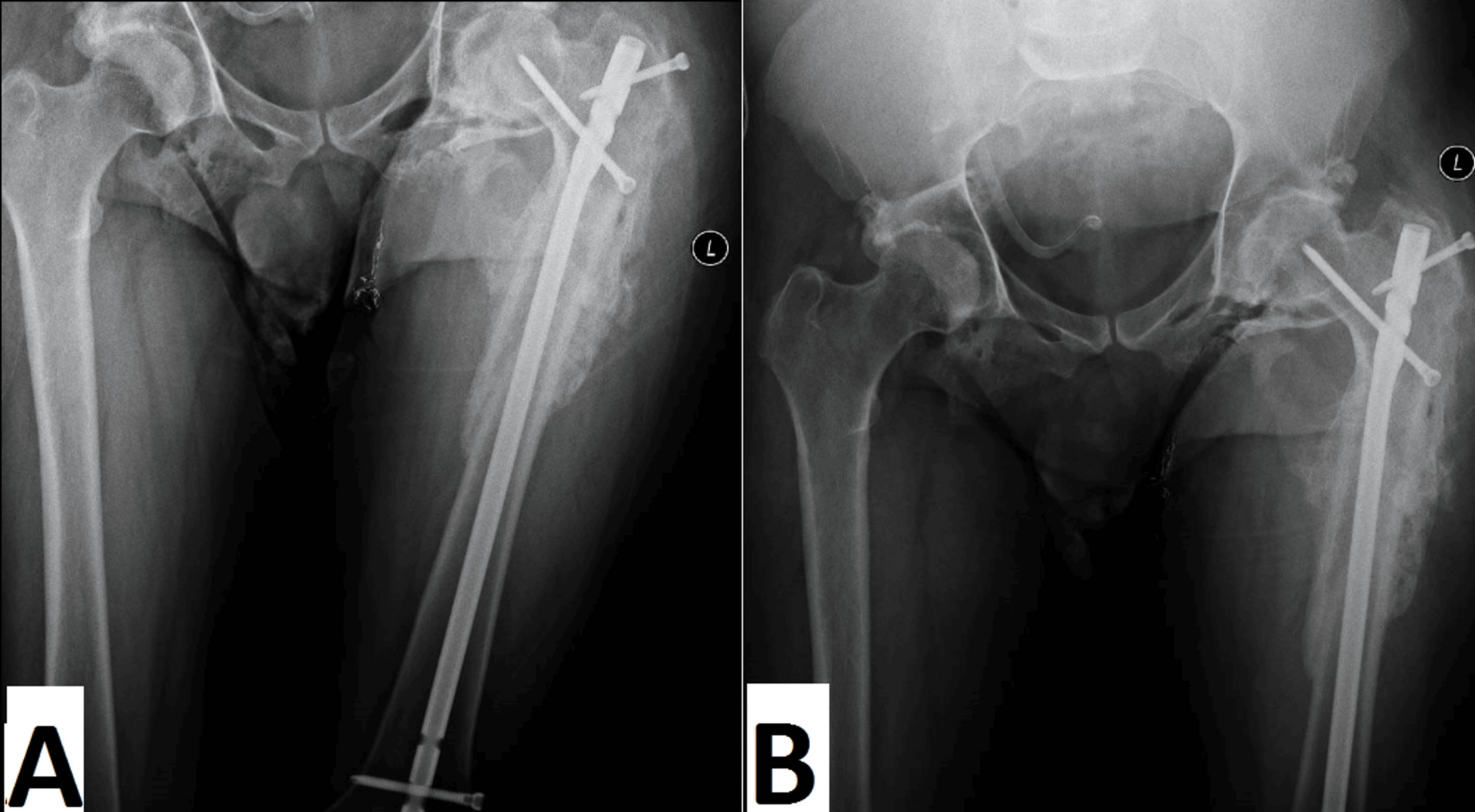
Follow-up plain X-ray, four months postoperative, confirms complete fracture healing.

The stitches were removed, and the left-side bed sore healed within two weeks. By four months postoperatively, the patient had successfully recovered to his pre-injury state. Follow-up plain X-rays confirmed complete fracture healing (Figure 7).

## Discussion

Osteoporosis is linked to spinal cord injuries, making patients more vulnerable to fractures from minor stress that may occur during routine activities like getting out of a wheelchair or getting into bed. Selecting the appropriate intervention methods poses a significant challenge and, because there have been few reported cases in the literature, an optimal treatment protocol has not yet been established. These patients are at a significant risk of experiencing fractures even with low-energy activities such as transferring or repositioning the patient due to several factors, including decreased bone quality, reduced muscle mass, and a thin insensate soft-tissue layer. Consequently, even minor trauma or routine activities can result in fractures that present significant treatment challenges, as demonstrated in our case [5].

Spasticity, an abnormal increase in muscle tone, can lead to deformities such as muscle contractures, joint contractures, torsional bone deformities, and joint instability. Various approaches to manage spasticity include physical therapy, passive stretching, casting of the extremities, medications that help reduce spasticity such as intrathecal baclofen, injections of botulinum toxin A (BTX-A), selective posterior rhizotomy (SPR), and surgical interventions. Orthopedic surgery should be considered a significant complementary intervention [6]. A 33-year-old man was diagnosed with a proximal femur insufficiency fracture after experiencing a sudden audible click while repositioning in bed, which resulted in left lower limb pain and deformity. The patient had a history of thoracic vertebral fractures of T7, T8, T9, and T10 from a motor vehicle accident and spastic paraplegia for 15 years. These fractures could result in significant complications for the patient [7]. Fracture characteristics and patient-specific factors play a crucial role in deciding whether surgical management is appropriate. Difficulties such as thinning of the soft tissue covering the fracture site and weakened bones due to osteoporosis often lead surgeons to consider nonsurgical approaches. Various methods have been employed for the nonsurgical treatment of fractures in patients with paraplegia. However, early interventions using skeletal and skin traction have shown unfavorable outcomes and increased complication rates [8].

Following open treatment, all patients in this group were able to return to their previous employment, regain their prior level of activity, and participate in competitive events. The goal of treatment is to promote healing while preserving the patient's functional ability. However, without proper care, the risk of complications is high, and the outcomes can be devastating. Consequently, our patient was treated surgically to reduce the risk of skin breakdown. Difficulties in sitting and muscle contractures are frequently encountered in adults with severe spastic paraplegia. The procedure was successful as well in improving the patient’s ability to sit despite the challenges faced in the fixation of this type of fracture in a patient with long-standing degenerative changes in the hip joint. Moreover, the significantly dislocated fracture had been anatomically reduced at follow-up, as shown by radiographs (Figures 5A-5D). Our case report highlights the successful treatment of non-ambulatory individuals with severe spastic paraplegia using intramedullary nail repair for a proximal femoral fragility fracture. The patient’s perineal hygiene was compromised, and this method of treatment was found to be more hygienic, effective, and provided better perineal care.

The patient’s examination and laboratory tests resulted in a diagnosis of vitamin D deficiency with a level of 10 ng/ml and severe degenerative osteoarthritic hips that negatively impacted his neurological conditions. As a result, the patient was unable to fully extend his hip and knee joints, which posed challenges during positioning on the traction table and the intraoperative reduction of the fracture and insertion of the guide wire (Figure 4). This is attributed to several reasons, highlighting the disruption of the parathyroid hormone-vitamin D axis, which often results in lower levels of parathyroid hormone and vitamin D in SCI patients. Approximately 61% of SCI patients suffer from secondary osteoporosis as a result of SCI [8]. Vitamin D supplementation was included as an essential part of the patient's medication regimen during his hospitalization period and continued after discharge as well.

Various investigations and laboratory tests have been performed, including nutritional assessments such as prealbumin level, blood culture, total lymphocyte count, and inflammatory markers. Based on a multicenter study by Wong S et al [8], approximately 44% of SCI patients admitted to the SCI center were malnourished. Therefore, assessing the nutritional status of patients can aid in identifying those who may benefit from nutritional assessments and optimization. Furthermore, conducting nutritional assessments is useful for ruling out infection. Bacteriuria and ulcer infections are common in SCI patients [8]. In our case, the patient’s suprapubic catheter was recently replaced, marking a significant update after 14 years. Additionally, he had a history of bilateral severe ischial decubitus ulcers, increasing his susceptibility to infection. Individuals with SCI are often colonized with antibiotic-resistant bacteria, including methicillin-resistant *Staphylococcus aureus* (MRSA), which can complicate infection management and treatment. This colonization is common and poses challenges for infection management and treatment [8]. Hence, IV vancomycin was added to his medication list during the postoperative period, and its level was monitored every other day. In patients with objective findings of infection, certain tests can be helpful. These include a complete blood count, urine and blood cultures, as well as inflammatory markers, such as the erythrocyte sedimentation rate and C-reactive protein level. These tests provide valuable information to diagnose and monitor infections in patients [9]. Physical therapy was initiated postoperatively to improve hip and lower extremity ROM. Despite the risk of fracture during simple activities, early intervention of physiotherapy using mobilization in patients with spinal cord injuries, using standing training or tilt table exercises, showed bone mineral density improvement. Physical therapy aims to utilize Wolff's law principles, which state that bones respond to mechanical stress to stimulate bone growth. However, it does not necessarily translate the increased bone density to increased strength and reduced fracture risk [10].

Hip fractures carry a high risk of mortality within the first year, with rates ranging from 12% to 36%. Additionally, only one-third of patients can regain their pre-fracture level of functioning, while another one-third require ongoing home care [11]. The present case showed complete recovery from the fracture (Figure 7A), and the patient returned to his pre-injury status after two years, which proved the success of the procedures we performed.

## Conclusions

Our case report highlights the successful treatment of non-ambulatory individuals with severe spastic paraplegia using intramedullary nail repair for a proximal femoral fragility fracture. The patient’s perineal hygiene was compromised, and this method of treatment was found to be more hygienic, effective, and provided better perineal care.

Subtrochanteric femoral fractures, especially in patients with spastic paraplegia, are rare and often unrecognized. Nevertheless, they constitute a surgical emergency. These fractures occur without any traumatic events or underlying pathology in individuals with spastic paraplegia. Therefore, it is crucial for medical professionals, physiotherapists, and caregivers to understand the important role of physiotherapy sessions and to handle patients carefully, avoiding any forceful movements. Further research is needed to determine the optimal treatment approach, including the potential role of soft tissue release in managing subtrochanteric insufficiency fractures.
